# Heparin, Heparin-like Molecules, and Heparin Mimetics in Breast Cancer: A Concise Review

**DOI:** 10.3390/biom15071034

**Published:** 2025-07-17

**Authors:** Diego R. Gatica Portillo, Yishu Li, Navneet Goyal, Brian G. Rowan, Rami A. Al-Horani, Muralidharan Anbalagan

**Affiliations:** 1 Department of Structural and Cellular Biology, School of Medicine, Tulane University, New Orleans, LA 70112, USA; dgaticap@tulane.edu (D.R.G.P.); ylee13@tulane.edu (Y.L.); browan@tulane.edu (B.G.R.); 2 Department of General Surgery, Taihe Hospital, Hubei University of Medicine, Shiyan 442000, China; 3 Department of Chemistry, Xavier University of Louisiana, New Orleans, LA 70125, USA; ngoyal@xula.edu; 4 Division of Basic Pharmaceutical Sciences, College of Pharmacy, Xavier University of Louisiana, New Orleans, LA 70125, USA

**Keywords:** heparin, heparin mimetics, LMWH, breast cancer, anti-metastatic, anti-angiogenic

## Abstract

Heparin and heparan sulfate are essential in various biological processes relevant to cancer biology and pathology. Given the clinical importance of breast cancer, it is of high interest to seek more effective and safer treatment. The application of heparins (UFH, LMWH, ULMWH, fondaparinux) and heparin mimetics as potential treatments is particularly interesting. Their use led to promising results in various breast cancer models by exhibiting anti-angiogenic and anti-metastatic properties. This article concisely reviews studies involving heparins and mimetics in both in vitro and in vivo breast cancer settings. We highlight molecules, conjugates, delivery systems, and combinations involving heparin or its mimetics. We also survey several potential biological targets such as VEGF, FGF-2, TGFβ-1, PDGF-B, NPP-1, CXCL12-CXCR4 axis, and CCR7-CCL21 axis. Overall, heparins and their mimetics, conjugates, and combinations represent a powerful strategy to effectively and safely treat breast cancer, which is the most common cancer diagnosed in women worldwide and the fifth leading cause of cancer-related deaths worldwide.

## 1. Introduction

Heparin/heparan sulfates (H/HS) belong to the superfamily of glycosaminoglycans (GAGs) and are linear polysaccharides composed of repeating disaccharide units of an amino sugar or uronic acid. They were first found in mucous membranes and mucosal secretions and hence have been named mucopolysaccharides. H/HS are highly charged due to the negatively charged carboxyl groups of uronic acid residues and sulfate groups on most units [1]. Notably, non-template-directed biosynthesis of H/HS allows for extensive modification like sulfation, acetylation, and epimerization, leading to substantial structural heterogeneity within each group [2].

The H and HS are key components of the proteoglycans and have closely related mechanisms of action. They are key players in numerous biological processes. At the cell surface and extracellular matrix, they enhance the structural integrity of cells and tissues. Electrostatic interactions between the negatively charged uronic acids and sulfate groups and the positively charged amino acids of proteins are mostly responsible for the capacity of H/HS to bind proteins. In addition, non-ionic interactions are essential in determining the selectivity and specificity of these binding interactions [3]. H/HS binds to cytokines, chemokines, growth factors, and enzymes and affects physiological processes, including coagulation, cell growth, infection, inflammation, tumor development, and metastasis. Heparin can be considered a specialized variant of heparan sulfate due to differences in sulfation, tissue specificity, and biological effects. Heparin is more sulfated and contains more iduronic acid than heparan sulfate, and it is produced primarily by mast cells [4,5]. In contrast, heparan sulfate is less sulfated with higher concentrations of glucuronic acid and is found on the cell surface of most cell types [4,5]. As stated above, both can induce a variety of biological effects; however, we can single out heparin’s and heparin variants’ biological effects as anti-coagulants. Heparin and its variants achieve their anti-coagulant properties through binding and activating their biological partner, antithrombin, to achieve much faster inhibition of thrombin (template- or bridging-based mechanism) and/or factor Xa (conformational change-based mechanism), two serine proteases in the common coagulation pathway. Several heparin variants, including unfractionated heparin (UFH), low molecular weight heparins (LMWHs), and fondaparinux, have long been clinically used as potent anti-coagulants. Heparan sulfate is much larger and less sulfated; thus, it is more important for a diverse array of biological functions beyond coagulation.

**Figure 1:**
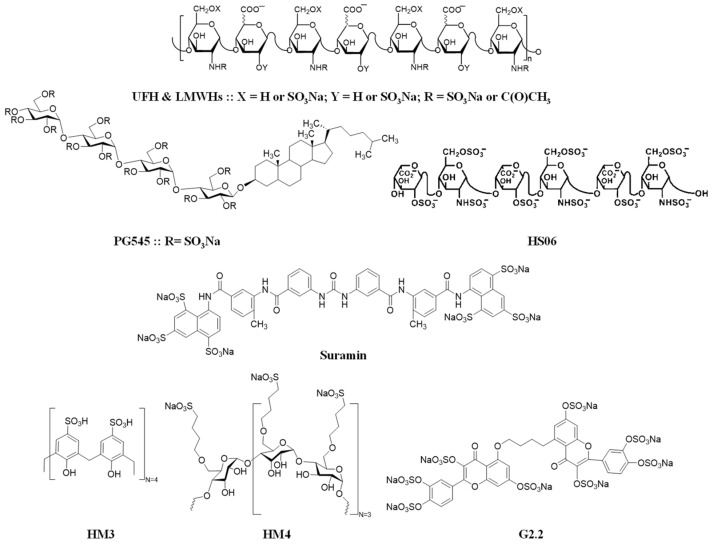
Chemical structures for heparin, heparin-like molecules, and heparin mimetics with potential application in breast cancer. Although subjective, the literature appears to define heparin-like molecules (top) as natural/synthetic polysaccharides with partial structural overlap, whereas heparin mimetics (bottom) are defined as synthetic/semi-synthetic compounds mimicking heparin’s sulfation patterns but structurally distinct (e.g., nonsaccharide scaffolds). The OSO_3_Na (sodium salt form) is the most predominant pharmaceutically available form. The OSO_3_^−^ (ionic form) is the biologically available form. These two forms are used interchangeably.

Because of their broad biological activities, H/HS and analogs have attracted significant interest in drug design for many diseases, such as cancer, inflammation, infection, wound healing, pulmonary disorders, and Alzheimer’s disease [6]. Here, we will review the role and therapeutic potential of heparin and its mimetics exclusively in cancer, focusing on breast cancer. In particular, H/HS derivatives (such as those in Figure 1) appear to be very relevant to cancer therapy, yet the bleeding risk in long-term use is a concern. Several H/HS-based molecules have been identified so as to retain anti-cancer properties while minimizing their anti-coagulation effects. These molecules were rationally designed by modifying the structure of heparins, particularly the antithrombin-binding site and other structural domains that are involved in interactions with cancer-related proteins. Structural design included changing the sulfation and/or *N*-acetylation patterns of heparins, adjusting the polysaccharide sequence length, attaching steroidal moieties, using the glycol-splitting strategies, or substituting the saccharide backbone with hetero-aromatic scaffolds.

## 2. Breast Cancer

Breast cancer is a pathology of paramount clinical importance, given that it is the most common cancer diagnosed in women worldwide and the fifth leading cause of cancer-related deaths worldwide [7,8]. Based on molecular and histological properties, breast cancer can broadly be divided into three groups: luminal-type (estrogen receptor/progesterone receptor-positive (ER+)), human epidermal growth factor receptor 2 (HER2) positive, and triple-negative [9]. ER+ type breast cancer represents breast cancers that are positive for the expression of estrogen receptors, progesterone receptors, or both. Depending on receptor positivity, hormones such as estrogen and progesterone can bind to their appropriate receptors and act to increase tumor growth in these cancers. ER+ breast cancer is the most common type of breast cancer, accounting for around 70–80% of breast cancer cases, and generally presents with a positive prognosis. In terms of molecular mechanisms in breast cancer, the estrogen receptor acts through two pathways, these being the classical (nuclear) pathway and the alternative (non-nuclear pathway). In the classical pathway, activated ER binds to specific DNA sequences called estrogen response elements (EREs). This binding regulates the expression of target genes, many of which are involved in processes like cell cycle regulation, differentiation, and angiogenesis. Some of these genes can promote cancer growth, highlighting the role of ER signaling in tumorigenesis. The alternative pathway occurs in the cytoplasm and refers to activation of coregulator growth factors and G-protein coupled signaling, including IGF-1 receptor, FGFR, HER2, mitogen-activated protein kinases, receptor tyrosine kinase, PI3K, AKT, mTOR, Src, and CDK [10]. Due to the mechanism involved with the pathology of ER+ breast cancer, targeted therapies exist that are tailored to the present receptors. Targeted endocrine therapies include SERMs (selective estrogen receptor modulators) such as tamoxifen, aromatase inhibitors, SERDs (selective estrogen receptor down-regulators) such as fulvestrant, and ovarian function suppression agents (through gonadotropin-releasing hormone agonists). Therapies which target the alternative pathways involved in ER+ breast cancer pathology have also been developed; these are increasingly relevant targets for ER+ breast cancer therapeutics due to de novo and acquired resistance of ER+ breast cancer to traditional targeted endocrine therapies. Such agents aim to inhibit PI3K, AKT, mTOR, and CD4/6 pathways at the G1/S checkpoint [10].

HER2 type breast cancer is a type of breast cancer in which there is overexpression/amplification of the human epidermal growth factor receptor 2 (HER2). HER2 is classified as a membrane tyrosine kinase, which is encoded by the ERBB2 gene, found on chromosome 17q12 [11,12]. This growth factor functions to regulate cell growth, and in HER2-type breast cancer, it promotes cancer cell proliferation and survival. HER2 accounts for around 15–20% of all breast cancers and is a more aggressive subtype compared to HER2-negative breast cancers [12]. Like ER+ breast cancers, targeted therapies have emerged that target the unique aspects of this subset of cancer. Targeted therapeutics against HER2 include monoclonal antibodies such as trastuzumab and pertuzumab. These targeted therapies have significantly improved patient outcomes and long-term survival [12].

Triple-negative breast cancer (TNBC) is a subtype of breast cancer that is negative for the estrogen receptor, progesterone receptor, and HER2. TNBC accounts for around 10–20% of breast cancer cases [13]. TNBC has been associated with BRCA1 mutations [13]. TNBC is considered an aggressive subtype of breast cancer, presenting clinically with higher-grade and increased size tumors, higher rates of recurrence, and higher rates of metastasis [14]. Due to these characteristics of TNBC, this subtype is associated with a poorer prognosis [14]. Due to the lack of potential endocrine targets, there are no targeted therapy options like there are for ER+ and HER2 breast cancer subtypes [13]. TNBC treatment mainly consists of chemotherapy as a neoadjuvant before surgery or as an adjuvant post-surgery; radiation therapy may also be indicated in certain cases [15]. Newer advances in TNBC treatment include immunotherapy through the anti-PD-1 agent pembrolizumab, PARP inhibitors such as olaparib, androgen receptor inhibitors, and PI3K/AKT/mTOR pathway blockers, amongst others [16,17]. It is important to emphasize here that certain common breast cancer cell lines used in vitro correspond to a specific breast cancer subtype. The MCF-7 cell line corresponds to the luminal-type, ER-positive subtype [18]. The MDA-MB-231 cell line corresponds to the triple-negative subtype [19]. The 4T1 cell line also corresponds to the triple-negative subtype [20].

A primary clinical concern in breast cancer is distal metastasis, which is the leading cause of mortality in the condition. Metastasis in breast cancer primarily occurs in the bones, lungs, liver, and brain [21]. Molecularly, breast cancer metastasis represents a complicated process with multiple biological factors involved in several mechanisms. For example, one of the factors involved in breast cancer metastasis is the CXCL12-CXCR4 axis, which facilitates tumor cell migration and invasion [22]. Given the clinical impact of breast cancer, it is of high interest to seek potential new avenues of treatment. One of these avenues is the application of heparin mimetics as a treatment, which has resulted in promising results in various breast cancer models by exhibiting anti-angiogenic and anti-metastatic properties. Conjugates of heparin or heparin mimetics with existing cancer therapeutic agents are also under study in the context of breast cancer, with favorable results.

## 3. Role of Heparin/Heparan Sulfate in Breast Cancer

Glycosaminoglycans (GAGs) are key players in cancer development, progression, and metastasis through their binding to growth factors, growth factor receptors, and cytokines. These bindings activate numerous signaling pathways essential for angiogenesis, tumor invasion, and metastasis dissemination. Notably, whereas some GAGs are involved in promoting cancer progression, others have been found to possess promising inhibitory effects on tumor growth. Advances in the knowledge of the multifunctional roles that GAGs play in many forms of cancer have prompted the development of novel therapeutic strategies and targeted drug therapies.

Specifically, heparin and heparan sulfate proteoglycans (H/HSPGs), via their core proteins and appended side chains, play a significant role in modulating a variety of functions of tumor cells and participate in tumor growth, invasion, and metastasis [23,24]. Studies have demonstrated that heparan sulfate facilitates cell–cell adhesion as well as cell–extracellular matrix adhesion, thereby preventing invasion and metastasis; however, reduced heparan sulfate expression, which is seen in certain cancers, leads to enhanced invasiveness of cancer cells [25,26]. While the levels of heparan sulfate decrease in some cancers, others show changes in their sulfation pattern, which are shown to be responsible for the development of cancer. HSPGs, such as syndecan-1 and syndecan-4, have also been involved in the development of breast cancer by interacting with fibroblast growth factors 1 and 2 and their receptors [27]. Other HSPGs of interest include the glypicans, cell surface HSPGs participating in cell development and signaling. Glypican-1 and glypican-6 are over-expressed in breast cancer cells, whereas glypican-3 is under-expressed [28].

Moreover, overexpression of heparanase (HPSE), a β-1,4 HS chain-cleaving enzyme of HSPGs, is involved in tumor growth-promoting mechanisms, angiogenesis, and metastasis. Heparanase inhibition is a potential target for cancer therapeutics since the action of heparanase leads to the degradation of the extracellular matrix and the release of angiogenic growth factors [29]. Heparin has previously been shown to function as a heparanase inhibitor, but due to undesirable anti-coagulant properties in cancer treatments, other heparin mimetics have been explored as heparanase inhibitors [29]. Multiple heparin mimetic compounds have been shown to exhibit heparanase inhibition, including PI-88, Suramin, PG545, PS3, JG3, STMCs, SST0001, and M402 [19]. Some of these compounds have seen application in breast cancer models, including suramin, PG545 (discussed later), and M402. M402, an HS mimetic glycol-split LMWH, has been shown to inhibit metastasis and prolong survival in an orthotopic triple-negative 4T1 breast cancer model [30]. Suramin was shown to be a potent in vitro growth inhibitor of both hormone-insensitive, estrogen receptor-negative human breast cancer cells (MDA MB231 and SK-BR-3) and hormone-responsive, estrogen receptor-positive human breast cancer cells (ZR 75-1, T47D, and MCF7). In MCF7 cells, suramin blocked the mitogenic action of growth factors such as epidermal growth factor (EGF) and insulin-like growth factors I and II (IGF-I and IGF-II, respectively), and it completely abolished the increase in cell proliferation induced by the steroid hormone 17β-estradiol. Suramin also significantly decreased the synthesis and secretion of the lysosomal enzyme cathepsin D, which was shown to be associated with a high risk of breast tumor metastasis [31]. Its activity was also studied in a pilot study of intermittent short infusions without adaptive control. The study indicated that suramin can be safely and conveniently administered to outpatients by intermittent infusion without using complex adaptive dosing strategies [32]. Several other studies report on suramin use in the context of breast cancer, suggesting the viability of heparin mimetics in this arena [33,34,35,36,37]. Several mechanisms were proposed for suramin’s anti-cancer activity, among which its anti-heparanase activity is gaining a great deal of interest [38]. Other important entities include human sulfatase-1 (SULF1) and human sulfatase-2 (SULF2). Downregulation of SULF1, a heparin-degrading endosulfatase, has enhanced the migration and invasion of breast cancer cells. Conversely, SULF2, an HS 6-O-endosulfatase, has been reported to inhibit tumor growth in vivo in human breast cancer xenograft models [39,40,41].

Additional research has focused on the interaction between heparin and other biological factors implicated in cancer pathologies. One such biological factor is the ectonucleotide pyrophosphatase/phosphodiesterase-1 (NPP1). Ectonucleotide expression is elevated in cancer settings, where they act to produce cancer-promoting adenosine. For example, high levels of NPP1 expression are associated with increasing bone metastasis in breast cancer. Researchers tested the effects of 15 heparin-related compounds on NPP1 activity, including UFH and LMWHs tinzaparin, enoxaparin, and fondaparinux. Researchers found UFH, fondaparinux, and tinzaparin to be the most potent inhibitors of NPP1, demonstrating complete NPP1 inhibition at specific concentrations. Moreover, the inhibition of NPP1 was highly selective, given that the inhibition of other ectonucleotides, such as NPP3 and NPP4, was minimally observed at whole NPP1 inhibition concentrations. The inhibition mechanism was shown to be allosteric through the computation of Lineweaver–Burk plots. Though current evidence highlights heparin compounds as promising therapeutic agents for reducing bone metastasis via their interaction with NPP1 [42], further investigation into the role of NPP1 in breast cancer is warranted.

More research on relevant biological factors has also investigated the role of the CCR7-CCL21 axis. The CCR7-CCL21 axis has been implicated in metastatic breast cancer pathology, particularly in metastasis to lymph nodes. It has been observed that the metastatic activity of CCL21 is dependent on binding to CCR7 and heparan sulfate. To illustrate this, researchers created a mutant CCL21 (mut-CCL21), which does not bind to heparan sulfate, and investigated its application in breast cancer metastasis. In preparation for this experiment, researchers analyzed levels of CCR7 expression in breast cancer cell lines, observing that CCR7 levels are mainly upregulated in the triple-negative 4T1 mouse mammary cancer cell line and to a less appreciable degree in the human triple-negative breast cancer MDA-MB-231 cell line. Application of mut-CCL21 in 4T1 and MDA-MB-231 cell lines resulted in decreased cell migration, including decreased trans-endothelial cell migration. In an in vivo tumor model of 4T1-Luc+ cells implanted into mice, mut-CCL21 application decreased luciferase expression in lymph nodes compared to control groups, pointing to anti-metastatic effects due to inhibition of lymph node spread. These results provide additional information on the role of heparan sulfate in the pathogenesis of metastatic breast cancer, substantiating the contribution of heparan sulfate’s interaction with the CCR7-CL21 axis [43].

## 4. Heparin and Mimetics as Anti-Breast Cancer Agents

The involvement of H/HS in cancer has been explored as a basis for developing novel therapeutics. Heparin, a naturally occurring sulfated GAG, is clinically used as an anti-coagulant to treat conditions such as thrombosis, thrombophlebitis, and embolism [44,45]. In addition to its anti-coagulant properties, heparin has been observed to display anti-cancer properties through anti-angiogenic and anti-metastatic effects [46]. Nevertheless, heparin’s high risk of bleeding complicates its use as an anti-cancer agent. The high bleeding risk of heparins is attributed to their ability to activate antithrombin, and subsequently to inhibit thrombin and factor Xa. Therefore, several heparin-like molecules and heparin mimetics have been developed to retain anti-cancer properties while minimizing their anti-coagulation effects. Accordingly, multiple cancer models have studied heparin-like molecules and heparin mimetics as potential treatments. Various cancers can present with bleeding concerns, making the anti-coagulant properties of heparin undesirable in long-term cancer treatment. To this end, various heparin mimetics have been developed that preserve the anti-cancer properties of heparin without exhibiting the anti-coagulant properties [28]. The bleeding/excessive anti-coagulant risk of many of these variants was assessed by measuring antithrombin (anti-IIa) and anti-factor Xa activity (anti-Xa) in human plasma, the effect on the activated partial thromboplastin time (APTT) and prothrombin time (PT) of human plasma as well as the bleeding times in animal models, such as the tail bleeding time in mice or rats.

In 2017, Afratis and co-workers described heparin and various heparin mimetics with observed anti-cancer effects, specifically in breast cancer models [28]. Treatment with heparin and heparin mimetics in breast cancer models has been associated with inhibition of cancer-promoting pathways. For instance, it has been shown that heparin treatment in MDA-MB-231, a triple-negative breast cancer cell line, and MCF-7, an estrogen receptor-positive human breast cancer cell line, downregulates pathways such as the RAF/MEK/ERK, PI3K/Akt, and TGFβ/SMAD (in MCF-7) pathways [47]. Heparin and heparin mimetics also display anti-metastatic properties in breast cancer models. LMWH treatment on an MDA-MB-231 cell line model reduced breast cancer cell invasion and migration by inhibiting tissue factor expression [48]. Another mechanism of anti-metastatic properties is the effects of heparin and heparin mimetics treatment on the C-X-C motif ligands, which are a subfamily of chemokines involved in immune cell migration and other biological processes. In an experiment using human breast cancer cells, heparin in the presence of IFN-gamma reduced the secretion of CXCL9 and CXCL10 (chemokines secreted by IFN-gamma). It inhibited IFN-gamma signaling, displaying anti-inflammatory effects [49]. Another process by which heparin and heparin mimetics are implicated in anti-cancer effects is inhibiting various growth factors, resulting in anti-angiogenic properties. LMWH conjugate LHT7 has inhibited VEGF-dependent phosphorylation in MDA-MB-231 breast cancer cells [50]; LHT7 has also been shown to block other factors such as fibroblast growth factor 2 (FGF2) and platelet-derived growth factor B (PDGF-B) [51].

Follow-up studies by the same research group used the heparin mimetic LHTD4, which was made by coupling LHT7 to a tetramer of deoxycholic acid (DOCA), with LHTD4 being combined with deoxycholylethylamine (DCK) for absorption enhancement. LHTD4 was found to be able to bind to TGFβ-1, which inhibited TGFβ-1 signaling, resulting in the suppression of TGFβ-1-related metastasis in a triple-negative MDA-MB-231 cell line model. In MDA-MB-231 cells, LHTD4 blocked CXCL12-induced CXCR4 phosphorylation, inhibiting the CXCL12-CXCR4 axis and its effects on metastasis. In an in vivo triple-negative 4T1 breast cancer model, LHTD4 was seen to inhibit the rate of metastasis and decrease tumor burden. An in vivo model of triple-negative MDA-MB-231 cells implanted onto the mammary fat pad of SCID mice and applied LHTD4 therapy orally showed decreased tumor burden across various organ sites of metastasis. Further, LHTD4 testing with LHTD4 treatment orally administered for 60 days at a 5 mg/kg/day dose on a mouse model of triple-negative MDA-MB-231 cells implanted into nu/nu mice found a significant reduction in lung metastases, with researchers correlating this to reduced CXCL12-CXCR4 axis activity due to observed lack of fibronectin expression (a marker for CXCL12-CXCR4 activity) in analyzed lung tissues [52].

Another heparin compound investigated is a nano-heparin derived from *Styela plicata*. The application of this compound was beneficial in a triple-negative MDA-MB-231 breast cancer cell model, suppressing invasion, proliferation, and proteasome activity and downregulating several extracellular matrix-related genes [53]. In 2020, Atallah and colleagues described novel heparin mimetic compounds and their applications to the breast cancer field [46]. PG545, a fully sulfated heparan sulfate mimetic, is one such compound that has demonstrated anti-tumor and anti-metastatic effects [54]. PG545 has been previously shown to inhibit heparanase activity and growth factors VEGF and FGF-2, inhibiting heparanase expression [35]. In a triple-negative 4T1 mouse breast cancer model, PG545 treatment administered in varying concentrations orally according to a treatment schedule was found to reduce both tumor growth and the number of lung metastases, as well as improve survivability. PG545 treatment was also found to inhibit the expression of heparanase in the tumor tissue of the 4T1 model [55].

Other heparin mimetics have also been found to have promising anti-tumor and anti-metastatic effects [56]. Several models studying metastatic breast cancer demonstrated these effects in the setting of metastasis to bone, a common site of breast cancer metastasis. TGF-β is involved in the pathogenesis of bone metastasis, promoting tumor cell growth and IL-11 levels, which promotes osteolytic lesions. One study applied the compound high-molecular-weight E. coli K5-derived heparin-like polysaccharide (UFH K5-NSOS) to a triple-negative MDA-MB-231 breast cancer cell model, wherein UFH K5-NSOS reduced IL-11 levels through TGF-β inhibition [57]. UFH K5-NSOS was also applied in a breast cancer bone metastasis model consisting of triple-negative MDA-MB-231 cells inoculated into mice’s left cardiac ventricles. UFH K5-NSOS (administered i.v. 5 mg/kg diluted in sterile water) was compared to Fragmin (LMWH, administered s.c.) and vehicle groups. UFH K5-NSOS had positive therapeutic effects by reducing weight loss and osteolytic lesion area [57]. Anti-coagulation activity of fragmin, heparin, and K5-NSOS was assessed by measuring antithrombin (anti-IIa) and anti-factor Xa activity (anti-Xa). K5-NSOS showed moderate anti-IIa and anti-Xa activity; in comparison, fragmin and heparin showed higher anti-IIa and anti-Xa activity. The effects of K5-NSOS and fragmin on APTT were also evaluated; K5-NSOS was found to have a weaker prolonging effect on APTT in comparison to fragmin. Through these results, researchers concluded that K5-NSOS has lower anti-coagulant properties in comparison to other heparin forms, such as traditional heparin and fragmin [57].

Likewise, Sai-Nan et al. identified multiple heparin mimetics used in breast cancer models [22]. A heparin-like compound of interest identified was LHbisD4, a conjugate of LMWH and four bis-deoxycholates. LHbisD4 was found to inhibit VEGF-C-induced phosphorylation of VEGFR-3, which resulted in the inhibition of VEGFR-3 effects in human dermal lymphatic endothelial cells. Researchers used a mouse model in which triple-negative 4T1 breast cancer cells were injected into the mammary fat pad of BALB/cSlc-nu mice. LHbisD4, administered orally, was assessed as a treatment for this mouse model. LHbisD4 was found to significantly reduce metastasis to lymph nodes, liver, brain, and bones. Axillary lymph nodes markedly decreased in volume with LHbisD4 treatment (compared to saline), showing LHbisD4’s potential to inhibit lymph node metastasis. A related experiment using triple-negative MDA-MB-231 human breast cancer cells demonstrated similar results of metastasis reduction to various organs and axillary lymph nodes, with lung metastasis reduction also observed with the MDA-MB-231 model and LHbisD4 treatment delivered orally [58]. Researchers attributed the anti-metastatic effects of LHbisD4 to its inhibitory effect on the VEGFC/VEGFR-3 axis [58]. Researchers have previously characterized the anti-coagulation activity of LHbisD4, finding by a factor Xa activity assay that LMWH conjugated with bis-deoxycholates (LHbisD4) exhibits decreased anti-coagulation effects [58].

Interestingly, HS06, a hexasaccharide sequence of HS, was found to have anti-cancer properties against cancer stem cells (CSCs) by inhibiting these cells’ capacity for self-renewal in various cancer cell lines, including breast triple-negative MDA-MB-231 cells. Researchers concluded that the inhibitory effects of HS06 are due to the activation of p38 MAP kinase (MAPK), with this activation causing the inhibition of the TCF4 signaling pathway, which serves as a regulator of CSC self-renewal (including breast CSCs) [59]. Follow-up studies by the same group have used the HS06 mimetic G2.2, which inhibited colon CSCs by activating p38 MAPK and was seen to enhance p38 in triple-negative MDA-MB-231 breast cancer spheroids [60].

Other studies investigating applications of heparin on breast cancer and related chemokines found that heparin dodecasaccharides represent the minimal chain length to bind and inhibit CXCL12 effectively. This inhibition affected metastatic potential in a triple-negative MDA-MB 231 breast cancer cell model [61]. Likewise, a study comparing heparin dodecasaccharides and heparin both administered through subcutaneous injection on an SCID mouse model of human breast cancer found that heparin dodecasaccharides reduced tumor lesion area but not the number of lung metastases. In contrast, heparin reduced both the tumor lesion area and the number of lung metastases [61].

More studies have investigated the applications of LMWH and its conjugates in treating breast cancer. A particular study examined the relationship between the Von Willebrand factor (VWF), cancer metastasis, and LMWH treatment. Researchers have previously associated VWF and metastatic breast cancer, observing that elevated VWF: Ag levels correlate with disease severity [62]. Increased endothelial cell activation in triple-negative MDA-MB-231 and estrogen receptor-positive MCF-7 breast cancer cells appears to result in elevated VWF release, which was attributed to the excessive secretion of VEGF-A by the cell lines studied. Applying LMWH to these cell models inhibited VWF levels and trans-endothelial migration, likely because of the inhibition of VEGF-A by LMWH. These results suggest a role of VWF secretion in tumor metastasis and LMWH as a potential avenue to target VWF-related metastatic breast cancer [62]. Demonstrating the same therapeutic potential, LMWH bemiparin and ultra-low-molecular-weight-heparin (ULMWH) RO-14 were found to inhibit cellular migration-related factors, capillary-like tube formation, and endothelial migration in a dose-dependent fashion. Both compounds also exhibited anti-angiogenic properties related to VEGF and FGF-2 activity [63]. Table 1 summarizes the primary anti-cancer effect of some of the described molecules.

## 5. Combinations and Delivery Systems Involving Heparin and Mimetics in Breast Cancer

Co-delivery systems consisting of heparin or heparin–mimetics in combination with traditional cancer therapeutic agents have seen extensive usage in breast cancer models. Heparin and its related compounds have many desirable characteristics for drug delivery systems, including excellent biocompatibility and binding ability to multiple compounds (i.e., other cancer therapeutic agents) [64,65].

One approach to co-delivery systems with heparin is the development of modified liposomes for treatment delivery. Liposomes have desirable characteristics in drug delivery, including biocompatibility and feasible modifications [66]. In one such study, alendronate-modified LMWH coated on the surface of doxorubicin (DOX)-loaded liposomes (A10-L-DOX-Lip) was developed and evaluated as a treatment. A10-L-DOX-Lip was found to more significantly inhibit in vitro cell migration and invasion in K7M2 and triple-negative 4T1 breast cancer cells than DOX-lip and free DOX groups, indicating the enhanced anti-metastatic effects via the inclusion of LMWH in the compound. A10-L-DOX-Lip (5 mg/kg, injection by vein tail) application to an in vivo breast cancer bone metastasis model showed strong anti-tumor effects, resulting in the smallest tumor sizes compared to other free DOX and DOX-lip treatment groups. Reduced bone resorption in the A10-L-DOX-Lip model was also observed, likely due to the actions of alendronate [64]. Prior work by the same research group used LMWH–cholesterol (LHC) conjugates with DOX in a triple-negative 4T1 breast cancer cell line model. The resulting DOX/LHC nanoparticles exhibited longer circulation time than DOX alone. In 4T1 cells, they were shown to be taken up more effectively and exhibited more significant anti-metastatic effects [67].

Another study utilizing liposomes developed photosensitizer indocyanine green (ICG) loaded into LMWH-modified liposomes, referred to as LMWH-ICG-Lip. This combination improved phototherapeutic efficacy by increasing the photosensitizer’s circulation time while lowering the risk of tumor metastasis by LMWH. In vivo, triple-negative 4T1 mice models showed greater biodistribution within tumor tissue for LMWH-ICG-Lip treatment groups (injected via the tail veins) than free ICG. Importantly, it was found that platelet aggregation takes place on tumor cells to enable immune evasion and increase metastasis. This is particularly true in hypoxic states. However, platelet adherence was inhibited in the LMWH-ICG-Lip treatment group, which was attributed to LMWH’s capacity to bind to P-selectin. Furthermore, 4T1 mice models studied for therapeutic efficiency showed decreased lung metastatic lesions in LMWH-ICG-Lip treatment groups [68].

A study utilizing micelles instead of liposomes used an a-tocopherol (TOC) and heparin polymeric micelle loaded with docetaxel (DTX) in a breast cancer cell model. Researchers found that DTX-loaded micelles had higher breast cancer cell toxicity than DTX alone. This suggested that heparin-modified micelles are a potential avenue for increased drug delivery of DTX in cancer treatment [69]. Another study examining the combination therapy of LMWH and chemotherapeutic agent adriamycin (Doxorubicin) used a C3H breast cancer model (C3H mice injected in the right axillary region with C3H breast cancer cells). The study included a normal saline group (administered intraperitoneally, once daily, 1 mL/20 g), an adriamycin group (administered intraperitoneally, 4 mg/kg/dose, once weekly), an LMWH group (administered subcutaneously, 1500 U/kg/day, once daily), and a combination group of LMWH/adriamycin (administered as previously described, in same concentrations). Combination therapy of LMWH/adriamycin was found to inhibit tumor growth, inhibit lung metastasis, promote tumor cell apoptosis, and inhibit VEGF expression [70]. Researchers speculate that LMWH’s mechanisms of anti-cancer effects may be through the upregulation of cancer cell apoptosis, inhibition of VEGF, and competition with heparanase for the HS acceptor/interactor that would lead to decreased metastasis through extracellular matrix integrity maintenance [70]. Also, a delivery system consisting of curcumin and paclitaxel co-loaded heparin (HSP-CUR-PTX) has been developed. Results show lower cytotoxicity compared to only PTX and increased inhibitory effect in estrogen receptor-positive MCF-7 breast cancer cell model compared to HSP-PTX treatment [70].

An oleanolic acid derivative (containing a quaternary ammonium cation moiety—QDT)/heparin/chitosan nanoaggregate (QDT/HEP/CS) was also considered for breast cancer treatment. Oleanolic acid has previously been shown to exhibit anti-cancer effects, and heparin and chitosan were used to create a drug delivery system. In an in vitro triple-negative 4T1 breast cancer cell model, QDT/HEP/CS treatment achieved a more significant anti-tumor effect, increased apoptosis (in a dose-dependent manner), and inhibited cell migration (in wound closure assay) compared to QDT alone. In an in vivo triple-negative 4T1 breast cancer tumor model, QDT/HEP/CS treatment (administered intraperitoneally, equal to 10, 20, 40 mg/kg QDT, every two days) prolonged retention time in tumor tissues, substantially inhibited tumor growth, and increased apoptotic induction compared to QDT treatment alone. Immuno-histochemistry on tumor tissue revealed increased pro-apoptotic factors caspase-3, caspase-9, and cytochrome C [71].

Efforts also included investigating the use of small extracellular vesicles (sEVs) from heparin-treated cells. sEVs, formerly exosomes, are produced by many cell types (epithelial cells and tumor cells) and act as agents of intercellular communication. sEVs contain biological factors such as proteins, mRNA, and miRNA, which are based on the tissues from which the sEVs are produced. This study isolated sEVs from the culture medium of estrogen receptor-positive MCF-7 and triple-negative MDA-MB-231 breast cancer cell lines treated with heparin, sEV-HT. Treating MCF-7 and MDA-MB-231 breast cancer cell models with sEV-HTs yielded similar results to isolated heparin(s) treatment (s). Markers such as miRNA 10b, miRNA21, miRNA23a, miRNA125b-1, miRNA137, and miRNA 145 were upregulated, while miRNA155 was downregulated. Levels of p-Akt and p-ERK were also downregulated, while levels of p-p53 and p21 were upregulated. Researchers concluded that sEVs are long-term reservoirs for heparin delivery [72]. The development of sEVs with heparin’s therapeutic properties represents an exciting area of future research, given the promising results observed.

## 6. Concluding Remarks and Future Directions

**Figure 2:**
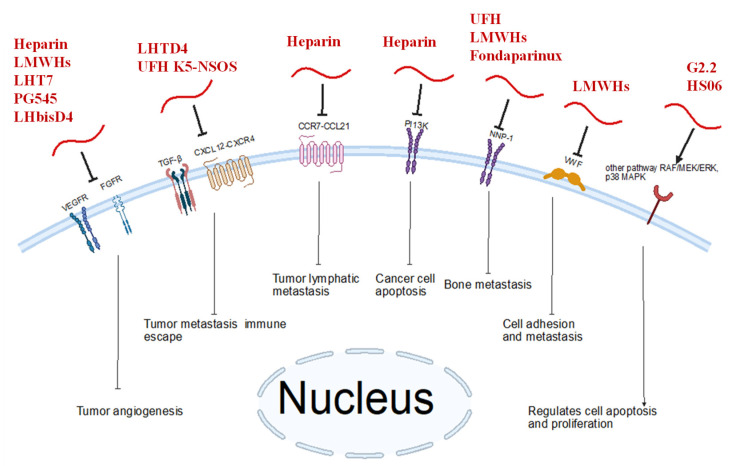
Molecular effects of heparin mimetics on various biological targets. Heparin mimetics can interact with a variety of biological targets, including VEGFR, FGFR, the CXCL12-CXCR4 axis, and others. The red thread represents heparin mimetics. Representative examples are provided.

As evidenced by several studies, there is clear potential for heparin mimetics (Figure 1) to have therapeutic potential in breast cancer settings. Heparin and heparin mimetics have shown significant anti-angiogenic and anti-metastatic effects in a wide variety of breast cancer models, as well as substantial benefits as part of co-delivery systems. Many of the heparin mimetics discussed in this review achieve anti-angiogenic effects through blockage of factors such as VEGF (including a variety of related factors such as VEGF-C/VEGFR-3 axis, VEGF-A), FGF-2, TGFβ-1, PDGF-B, and NPP-1. Anti-metastatic effects observed may be achieved through the blockage of the CXCL12-CXCR4 axis. Other factors relevant to breast cancer metastasis are potential future targets for inhibition, such as the CCR7-CCL21 axis. Figure 2 illustrates the multiple biological targets that heparin mimetics are able to interact with, and the subsequent molecular effects seen. Table 1 further specifies the anti-cancer effects of specific heparin mimetic molecules, since these effects vary depending on the heparin mimetic.

Anti-angiogenic and anti-metastatic effects were observed in varied breast cancer cell lines, including human estrogen receptor-positive MCF-7, human triple-negative MDA-MB-231, and mouse triple-negative 4T1. Given the multiple cell lines studied, we conclude that heparin mimetics have therapeutic potential in multiple breast cancer subtypes, including at least estrogen receptor-positive types and triple-negative types. Since the observed effects of heparin mimetics depend on interactions with a variety of growth factors as well as pro-metastatic biological factors (such as the CXCL12-CXCR4 axis, CCR7-CCL21 axis), it is possible that these angiogenic and metastatic factors are common to the majority of breast cancer types, thus making heparin and heparin mimetics potential therapeutic agents for most types of breast cancer.

In addition to the mimetics mentioned above, new cyclic, saccharide, and nonsaccharide heparin mimetics are being investigated for applications in breast cancer models. HM3 and HM4, heparin mimetics initially developed to be safer and more selective anti-coagulants [73,74], were applied as concomitant treatments in a triple-negative MDA-MBA-231 and estrogen receptor-positive MCF-7 breast cancer cell models in our lab. HM3 and HM4 significantly reduced the number of cells in both cell lines post-treatment compared to vehicle controls. The two molecules represent a new generation of cyclic heparin mimetics, synthetically feasible in 3–5 chemical steps. They have no demonstrated effect on human plasma clotting times of APTT and PT at concentrations up to 500 µM. Furthermore, they exhibited no cellular toxicity in MTT assays at similar concentrations. HM3 and HM4 are currently under extensive mechanistic studies and in vivo testing, yet initial results suggest a paradigm-shifting results.

A limitation of the present body of work concerning heparin mimetics application in breast cancer therapy is the limited number of in vivo studies that have been undertaken. Heparin mimetics tested in in vivo conditions include LHTD4, PG545, UFH K5-NSOS, LHbisD4, heparin dodecasaccharides, A10-L-DOX-Lip, LMWH-ICG-Lip, LMWH/Adriamycin, and QDT/HEP/CS. These compounds have demonstrated varied anti-cancer effects in vivo through anti-tumorigenic and anti-metastatic effects. There is a limited number of clinical trials in this arena [75]. More clinical trials are needed to see if the anti-cancer effects of heparin mimetics observed in vitro and in vivo can be reliably reproduced in human clinical settings. It is also important to conduct thorough pharmacokinetic profiling, toxicity studies, and address regulatory challenges before these agents can be introduced into clinical settings.

## Figures and Tables

**Table 1:** Anti-cancer effects of various relevant molecules.

Molecule	Primary Anti-Cancer Effect
LHT7	Inhibits VEGF-dependent phosphorylation in MDA-MB-231 breast cancer cells. Blocks FGF2 and PDGF-B.
LHTD4	Inhibits TGFβ-1 signaling and CXCL12-induced CXCR4 phosphorylation. Reduces metastasis in vivo.
Nano-heparin (*Styela plicata*)	Demonstrates inhibitory effects on invasion, proliferation, and proteasome activity in MDA-MB-231 cells.
PG545	Reduces tumor growth and lung metastases in a 4T1 mouse breast cancer model.
UFH K5-NSOS	Inhibits IL-11 levels via TGF-β inhibition. Reduces osteolytic lesions in breast cancer bone metastasis models.
LHbisD4	Inhibits VEGF-C-induced phosphorylation of VEGFR-3. Reduces metastasis to lymph nodes, liver, brain, and bones in mouse models.
HS06	Inhibits cancer stem cells (CSCs) by activating p38 MAP kinase. Suppresses self-renewal pathways in MDA-MB-231 breast cancer cells.
G2.2	Inhibits cancer stem cells by activating p38 MAPK.
Heparin	Reduces both tumor lesion area and the number of lung metastases in SCID mouse models of human breast cancer.
Bemiparin (LMWH) and RO-14(ULMWH)	Reduce endothelial angiogenic features, capillary-like tube formation, and cellular migration-related factors in breast cancer models.

## Data Availability

No new data are included.
